# FTO‐Catalysed Demethylation of LUR1 mRNA Suppresses Macrophage Lipid Accumulation and Aortic Atherosclerosis

**DOI:** 10.1111/jcmm.71234

**Published:** 2026-06-11

**Authors:** Xiang‐Yang Tang, Le Zhou, Shu‐Jun Li, Shao‐Xiang Zhang, Yue‐Ying Yuan, Yun‐Cheng Lv

**Affiliations:** ^1^ Guangxi Key Laboratory of Diabetic Systems Medicine & Cardiology Department of the Second Affiliated Hospital Guilin Medical University Guilin Guangxi China; ^2^ Gastroenterological Department The First Affiliated Hospital of Shaoyang University Shaoyang Hunan China

**Keywords:** atherosclerosis, FTO, lipid metabolism, LUR1, m6A

## Abstract

Lipid deposition and foam cell formation drive atherogenesis. Lipid uptake regulator 1 (LUR1) and N6‐methyladenosine (m6A) modification play key roles in systemic lipid homeostasis. Bioinformatic analyses revealed a negative correlation between fat mass and obesity‐associated protein (FTO) and LUR1 expression and abundant m6A sites within the LUR1 mRNA strand. We therefore hypothesized that FTO‐catalysed demethylation modulates LUR1 expression and lipid accumulation in macrophages, thereby influencing aortic plaque development. Acetylated low‐density lipoprotein‐loaded THP‐1 macrophages were transduced with recombinant lentiviruses, and FTO and LUR1 expression was determined by Western blotting and qPCR. m6A abundance was measured by methylated RNA immunoprecipitation–qPCR. Intracellular lipids were quantified by ELISA, and lipid droplets (LDs) were quantified using Oil Red O (ORO) staining. The plasma lipid concentration, aortic sinus plaque area and lipid deposition were evaluated by biochemical assays and histological staining in high‐fat‐fed apoE^−/−^ mice that received recombinant adeno‐associated viruses. The results revealed that elevated FTO expression not only reduced LUR1 expression and its m6A modification and decreased intracellular LD and cholesterol ester contents but also significantly decreased plasma triglyceride (TG) and low‐density lipoprotein‐cholesterol levels (LDL‐C) and decreased the aortic lesion area. Co‐overexpression of FTO and LUR1 abolished these protective effects. These findings indicate that FTO‐erased m6A modification decreases LUR1 expression in macrophages to inhibit intracellular lipid accumulation and the occurrence of atherosclerosis (AS).

**Abbreviations**
AAV: adeno‐associated viruses
ACAT2: acyl coenzyme A‐cholesterola‐cholesterol acyltransferase 2
ac‐LDL: acetylated low densitylow‐density lipoprotein
AS: atherosclerosis
CDK2: cyclin‐dependent kinase 2
FTO: fat mass and obesity‐associated protein
HDL‐C: high‐ density lipoprotein‐cholesterol
HFD: high‐fat diet
LD: lipid droplet
LDL‐C: low density lipoprotein‐cholesterol
LDLR: low‐density lipoprotein receptor
LUR1: lipid uptake regulator 1
m6A: N6‐methyladenosine
OE‐FTO: overexpression of FTO
OE‐LUR1: overexpression of LUR1
ORO: Oil Red O
PPARγ: peroxisome proliferator activated receptor‐γ
S1P: site‐1 protease
TC: total cholesterol
TG: triglyceride

## 1 Introduction

Atherosclerosis (AS) is the pathological foundation of cardiovascular diseases and is the leading cause of mortality worldwide [1, 2]. The formation of lipid‐laden foam cells within arterial walls constitutes the hallmark of early atherosclerotic lesions and drives plaque progression [3, 4]. Under proatherogenic conditions, monocytes infiltrate the subendothelial space, differentiate into macrophages and avidly internalize modified lipoproteins, ultimately leading to the transformation of these cells into cholesterol‐rich foam cells [5]. Consequently, suppressing macrophage lipid accumulation and foam cell formation represents a promising therapeutic strategy for atherosclerotic cardiovascular disease [4].

Lipid uptake regulator 1 (LUR1, also known as SPRING/POST1/C12orf49) has emerged as a novel regulator of lipid metabolism [6, 7]. As a Golgi membrane protein, LUR1 facilitates site‐1 protease (S1P) maturation, thereby activating the SREBP pathway to promote cholesterol uptake and fatty acid synthesis in hepatocytes [8, 9, 10, 11]. Notably, the role of LUR1 in macrophage cholesterol metabolism and atherogenesis remains entirely unexplored and represents a critical knowledge gap. Bioinformatic analyses using sRAMP and RMBase v2.0 predicted multiple conserved N6‐methyladenosine (m6A) modification motifs within the LUR1 mRNA transcript that were predominantly localized near exon–intron junctions. Importantly, these databases further indicated that these conserved sites represent potential binding targets for the m6A reader protein YTHDF1. Furthermore, GEO dataset analysis (GSE125771) revealed a significant negative correlation between the expression of LUR1 and that of FTO (an m6A demethylase) in human atherosclerotic plaques (R = −0.71). These observations led us to hypothesize that FTO‐mediated epigenetic regulation might downregulate macrophage LUR1 expression to inhibit lipid accumulation.

M6A modification represents the most prevalent internal chemical modification of eukaryotic mRNA and critically influences mRNA stability and translation efficiency [12, 13, 14]. The m6A demethylase fat mass and obesity‐associated protein (FTO) catalyses the removal of m6A marks, thereby modulating target gene expression and cellular lipid metabolism [15, 16, 17, 18, 19]. Beyond its established roles in adipogenesis and energy homeostasis, FTO has been implicated in cardiovascular protection: it attenuates vascular smooth muscle cell senescence to retard atherosclerotic plaque progression [20] and mitigates adverse cardiovascular remodelling [21]. Specifically, FTO‐mediated demethylation typically reduces the mRNA stability or translational efficiency of target transcripts, providing a mechanistic basis for its observed negative correlation with LUR1 in atherosclerotic lesions.

Given that current investigations have largely focused on hepatic lipid metabolism via the SREBP pathway [22, 23, 24], the comprehensive role of LUR1 in extrahepatic tissues—particularly in macrophage lipid homeostasis and atherogenesis—remains poorly characterized. Therefore, the present study investigated the regulatory role of LUR1 in lipid accumulation in macrophages and elucidated its upstream epigenetic mechanism, namely, FTO‐catalysed m6A demethylation. To this end, we first manipulated FTO and LUR1 expression in THP‐1 macrophages to establish their functional interplay in terms of intracellular lipid accumulation. We then combined bioinformatics analyses with targeted m6A detection assays to elucidate the underlying epigenetic mechanism. Finally, using high‐fat diet‐fed apoE^−/−^ mice, we confirmed that FTO‐mediated m6A demethylation suppresses aortic LUR1 expression and lipid deposition, thereby attenuating atherosclerotic lesion formation. These findings may provide a novel epigenetic strategy for the prevention and treatment of atherosclerotic cardiovascular disease.

## 2 Materials and Methods

### 2.1 Cell Culture

THP‐1 monocytes (a human acute monocyte leukaemia cell line) were obtained from the Chinese Academy of Sciences Cell Bank. The cells were grown in RPMI 1640 medium (Beijing Solarbio, 31,800,022, China) supplemented with 10% fetal bovine serum (FBS) (Thermo Fisher Scientific Inc., 10100147C, USA) and 1% penicillin–streptomycin (Biosharp, BL505A, China) in a humidified 5% CO_2_ atmosphere at 37°C. THP‐1 cells were differentiated into macrophages (hereafter referred to as ‘THP‐1 macrophages’) by incubating them with 150 nM phorbol 12‐myristate 13‐acetate (PMA; Sigma, P8139, USA) for 24 h, after which the culture medium was replaced with RPMI 1640 medium for 24 h. The differentiated phenotype was visually inspected under an optical microscope, after which the cells were rinsed two times with phosphate‐buffered saline (PBS), followed by an additional 48 h of incubation in RPMI 1640 medium with the experimental intervention as indicated below or together with 100 μg/mL human acetylated low‐density lipoprotein (ac‐LDL) when needed.

### 2.2 Animals and Treatments

Apolipoprotein E knockout (apoE^−/−^) mice (males, 6 weeks old) and a high‐fat chow (HFD) (Research Diets D12079B, 40% kcal from fat, 17% kcal from protein, 43% kcal from carbohydrate, 4.7 kcal/g, containing approximately 0.21% cholesterol; Nanjing Cavens) were purchased from Nanjing Cavens Laboratory Animal Co. Ltd. All the mice were housed in a specific pathogen‐free environment throughout the experiment. The AS model was established by administering a HFD instead of an ordinary diet for 1 week. The mice were randomly divided into the control group, AAV‐FTO group, and AAV‐FTO + AAV‐LUR1 group (6 for each). At the beginning of the 5th week, all the mice were treated with control or recombinant adeno‐associated viruses (AAV9 vector with a CMV enhancer‐driven promoter; Genechem Co. Ltd., Shanghai, China) to alter the expression of FTO and LUR1 in vivo. Each virus was injected at a dose of 1.0 × 10^11^ viral genomes (vg) per mouse via the tail vein. 4 weeks later, all the animals in each group were euthanized, blood was taken after eyeball extraction, the cavities of the thorax and abdomen were immediately opened, and their hearts were exposed to perfusion. The hearts and aorta were harvested for subsequent examinations (Ethical approval number: GLMC‐IACUC‐20251093).

### 2.3 Western Blotting

Western blotting was performed according to standard protocols. The cells were lysed in RIPA solution (Beijing Solarbio, R0010, China) supplemented with PMSF (Beijing Solarbio, P0100, China) (RIPA: PMSF = 94: 6) for total protein extraction. The collected proteins were separated by SDS–PAGE (Beijing Dingguo Changsheng Biotechnology, WB‐0201, China) and then transferred to PVDF (Merck Millipore, DVPP00010, China) membranes. The membranes were incubated with rabbit anti‐FTO (1:1000; Proteintech, 27,226–1‐AP; China), rabbit anti‐LUR1 (1:2000; Biorbyt orb459939; China) or rabbit anti‐GAPDH (1:8000; Proteintech, 60,004–1‐Ig; China) antibodies. Immunoreactive protein bands from at least 3 independent experiments were measured with an enhanced chemiluminescence immunoblotting detection system (Tanon 5500 Multi, Shanghai, China), and densitometry was performed using ImageJ software. Original uncropped Western blot images are shown in Files S1 and S3.

### 2.4 RNA Isolation and Quantitative Real‐Time PCR (qRT–PCR)

RNA was extracted using TRIzol Reagent (Sangon Biotech, B511311, China). cDNA was prepared by amplifying 500 ng of RNA with a SuperScript‐II cDNA Synthesis Kit (Sangon Biotech, B639277, China). Quantitative PCR was performed using a RevertAid First Strand cDNA Synthesis Kit (Sangon Biotech, B300538, China) with 1 mg of RNA, and qRT–PCR was performed using an UltraSYBR One Step RT–qPCR Kit (Sangon Biotech, B300540, China) following the manufacturer's instructions. The relative expression levels of FTO and LUR1 were normalized to those of GAPDH. Statistical significance was determined by one‐way ANOVA with Welch's correction using GraphPad Prism software. The primers used were as follows: FTO FOR: 5′‐ACTTGGCTCCCTTATCTGACC‐3′, FTO REV: 5′‐TGTGCAGTGTGAGAAAGGC TT‐3′, LUR1 FOR: 5′‐GGTCTGAGTCCCTCGCCTCCAGG‐3′, LUR1 REV: 5′‐GGATGAACTTAACCTTCCACTGG‐3′, GAPDH FOR: 5′‐ATCCCATCACCATCTT CC‐3′, and GAPDH REV: 5′‐GAGTCCTTCCACGATACCA‐3′.

### 2.5 The Quantification of Global m6A Level on Total mRNA

The level of m6A on total RNA was evaluated using a commercial m6A RNA methylation quantification kit (Abcam, ab185912, USA). First, total RNA was extracted from the sample, followed by DNase I administration to eliminate DNA contamination and RNA purification to obtain pure RNA samples. An m6A RNA methylation quantification kit was subsequently used according to the manufacturer's instructions. The concentration and purity of the samples were assessed by measuring the absorbance at 260 nm and 280 nm using a UV spectrophotometer. Finally, bioinformatics analysis was conducted to elucidate the relationship between m6A modification and biological function in the context of AS.

### 2.6 MeRIP‐qPCR

After the RNA lysate of treated cells was collected (RIP Lysis Buffer: Protease inhibitor: RNase Inhibitor = 100: 0.5: 0.25), an Imprint RNA immunoprecipitation kit (Sigma–Aldrich, RIP‐12RXN, USA) was used for RNA immunoprecipitation (300 μg total RNA for each immunoprecipitation). The Magna ChIP Protein A/G Magnetic Beads (Sigma–Aldrich, 16–663, USA) were prewashed and incubated with an anti‐m6A antibody (or with normal IgG as a negative control RIP reaction) for 0.5 h at 25°C with rotation. After 3 washes, the antibody‐conjugated beads were mixed with purified poly (A) RNA and 1 × immunoprecipitation buffer supplemented with RNase inhibitors at 4°C overnight. M6A‐containing mRNA was eluted twice with 20 mM m6A 5′‐monophosphate sodium salt. Afterward, the methylated mRNA was precipitated with glycogen and one‐tenth volume of sodium acetate in 100% ethanol at −80°C overnight. Further enrichment was calculated by qPCR, and the corresponding m6A enrichment in each sample was calculated by normalization to the input.

### 2.7 ORO Staining

Lipid droplets (LDs) were visualized by ORO staining. After being incubated with ac‐LDL together with the experimental treatments, the THP‐1 macrophages were fixed with 4% formalin (Servicebio, G1101, China) for 0.5 h, stained in prewarmed ORO solution for 1 h, and then stained with haematoxylin for 10 min. The stained cells were rinsed with double distilled water and then covered with an appropriate amount of 60% glycerol to avoid exposure to air. The red‐stained LDs were observed under a light microscope and further quantified with ImageJ (National Institutes of Health, Bethesda, MD, USA). For in vitro quantification, THP‐1 macrophages were seeded at a uniform density, and representative microscopic fields with comparable cell confluence were selected for imaging. ORO‐stained images were analysed using ImageJ software. The thresholding tool was applied to isolate red‐stained LDs. Lipid accumulation was quantified as the area fraction of LDs (positive staining area/total field of view) and is presented as the percentage of LDs (%).

### 2.8 Lipid Content Determination

The intracellular contents of total cholesterol (TC) were determined using a colorimetric assay and microplate reader to measure the optical densities of the sample tubes and the calibration standard tubes. The cell lysate supernatant was collected, purified and diluted with 20× washing buffer according to the instructions of the ELISA test kit (Yuanxin Biotechnology, YX‐SH‐A0161, China, diluted at a 1:19 ratio). The test samples and the different concentrations of standard samples were reacted with an HRP‐labelled cholesterol ester detection antibody for 1 h in a 37°C constant‐temperature incubator. After the reaction, the OD value of each plate well was determined from a spectrophotometer at 450 nm and used to calculate the content of TC in each test sample. Blood samples from each mouse were centrifuged at 4°C and 3000 rpm for 15 min, and the supernatant was collected for immediate testing. In accordance with the instructions of test kits (Yuanxin Biotechnology, YX‐SH‐A0161, China, diluted at a 1:19 ratio) for measuring triglyceride (TG), TC, high‐density lipoprotein‐cholesterol (HDL‐C), and low‐density lipoprotein‐cholesterol (LDL‐C), the corresponding parameters were set on a fully automatic biochemical analyser to measure the blood samples once the samples were loaded. The plasma lipid results were exported after the automatic biochemical analyser completed the measurement.

### 2.9 Histological Staining

After the experimental animals were sacrificed, the aortic roots were fixed with formalin and embedded in OCT matrix for frozen tissue sectioning. After the plane with three intact aortic valve leaflets was identified, the OCT‐embedded aortic root was cut into 8‐μm‐thick consecutive sections. A total of 15 consecutive sections were collected from the aortic root of each mouse, from which 3 sections were randomly selected as technical replicates for ORO and H&E staining. Plaque area and lipid deposition were quantified in ORO‐stained sections using Image‐Pro Plus 6.0 software (Media Cybernetics, USA). Additionally, the aortic arch and its three branches (brachiocephalic trunk, left common carotid artery, and left subclavian artery) were carefully isolated, opened longitudinally and pinned flat. The vessels were then subjected to en face ORO staining to evaluate the lesion area, which was quantified as a percentage of the total aortic surface area using Image‐Pro Plus 6.0 software.

### 2.10 Immunohistochemistry

To perform immunohistochemical determination, the frozen sections were removed from the 4°C refrigerator, and a hydrophobic circle was drawn around the tissue edge with an immunohistochemistry pen. After treatment with an endogenous peroxidase blocker and a nonspecific staining blocker, a diluted rabbit‐derived FTO antibody (1:100) was added, and the samples were incubated overnight at 4°C and then sequentially incubated with biotin‐labelled rabbit IgG polymer reagent and streptavidin‐horseradish peroxidase reagent for 10 min, respectively. The frozen sections were dyed with DAB solution and haematoxylin and then dehydrated and sealed with neutral resin for subsequent observation. Microphotographs were captured using a Zeiss microscope equipped with digital image processing software (AxioVision Imaging System, Germany).

### 2.11 Immunofluorescence

After the frozen sections were stored in a 4°C refrigerator, a water‐repellent marker pen was used to draw a circle around the tissue. The sections were then rinsed with double‐distilled water and PBS. Next, 10% goat serum was added to the circle to block the sections overnight at 4°C. The primary anti‐LUR1 antibody, sourced from rabbits, was diluted to a concentration of 1:50 and incubated at 4°C overnight. After being washed five times with TBS buffer, the secondary antibody was diluted according to the antibody manual and incubated at 37°C for 1 h. DAPI working solution was added to the circle and incubated for 10 min, followed by five gentle washes with TBS buffer. Finally, after the antifade mounting medium was added and the slides were sealed with neutral balsam, they were observed and evaluated under a fluorescence microscope. The entire procedure was carried out in the dark.

### 2.12 Statistical Analysis

Statistical analysis and graphing were performed using GraphPad Prism 10 software. All experiments were repeated three times, and the results are presented as the means ± standard deviations (SDs). The experimental data were analysed by one‐way analysis of variance (ANOVA) unless otherwise indicated. A *p* < 0.05 was considered to indicate statistical significance.

## 3 Results

### 3.1 FTO Inhibited Lipid Accumulation in Macrophages

**FIGURE 1:**
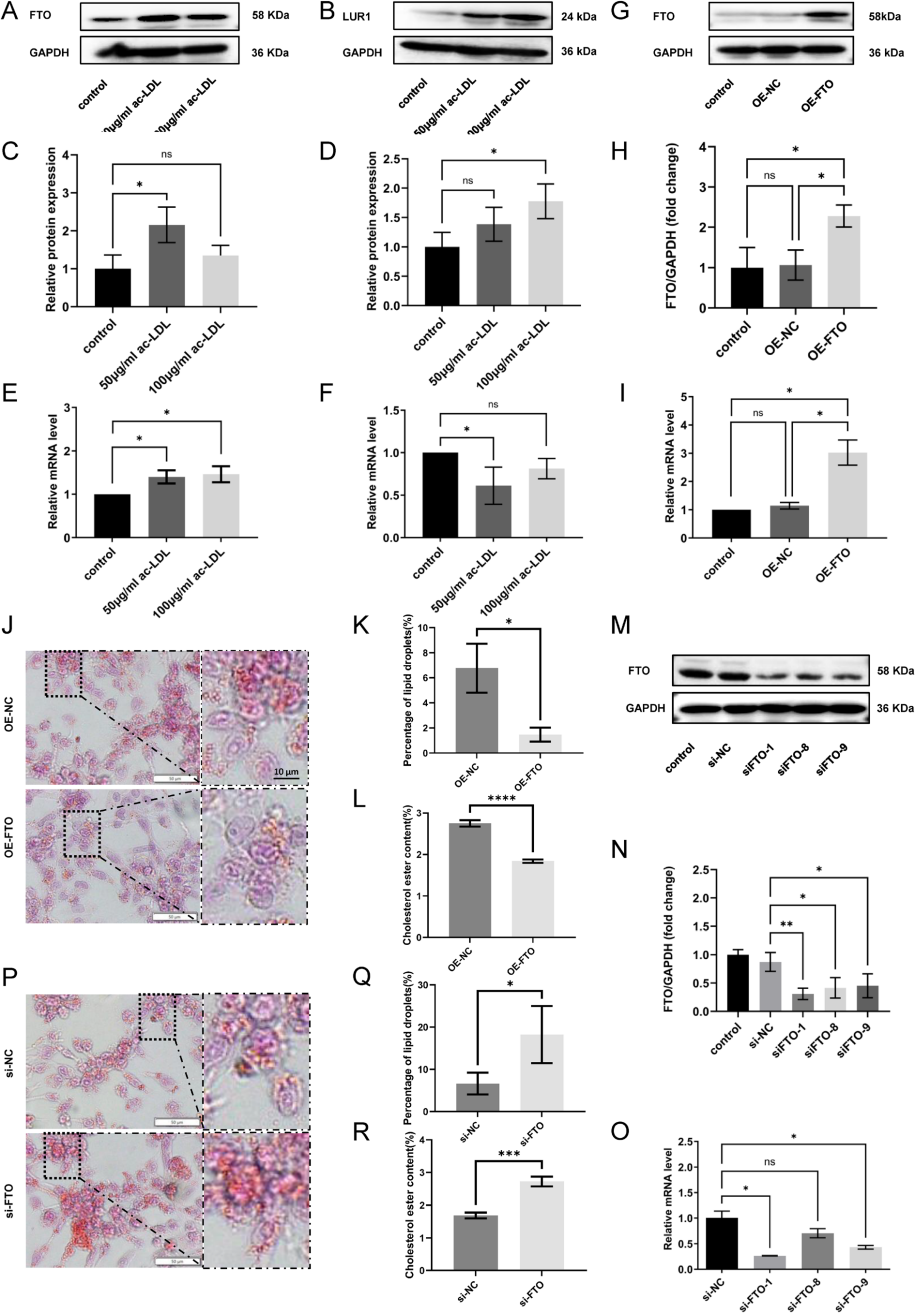
The suppressive effect of FTO on LUR1 expression and lipid accumulation in macrophages. (A–D) The protein levels of FTO and LUR1 in THP‐1 macrophages incubated with different doses of ac‐LDL for 48 h. (E–F) mRNA levels of FTO and LUR1 in THP‐1 macrophages incubated with different doses of ac‐LDL for 48 h. (G–H) FTO protein expression in THP‐1 macrophages transfected with OE‐FTO for 48 h. (I) The levels of FTO mRNA in THP‐1 macrophages transfected with OE‐FTO for 48 h. (J‐K) Intracellular LDs stained with ORO in THP‐1 macrophages transfected with OE‐FTO for 48 h (40×; inset shows a magnified view of representative LDs; scale bars, 50 μm [original] and 10 μm [inset]). (L) Intracellular TC content measured by ELISA in THP‐1 macrophages transfected with OE‐FTO for 48 h. (M, N) FTO protein expression in THP‐1 macrophages transfected with FTO siRNAs for 48 h. (O) The levels of FTO mRNA in THP‐1 macrophages transfected with FTO siRNAs. (P,Q) Intracellular LDs stained with ORO in THP‐1 macrophages transfected with siFTO‐1 for 48 h (40×; inset shows a magnified view of representative LDs; scale bars, 50 μm [original] and 10 μm [inset]). (R) The intracellular contents of TC measured by ELISA in THP‐1 macrophages transfected with siFTO‐1 for 48 h. All the results are expressed as the mean ± SD from three independent experiments in duplicate; *p < 0.05, **p < 0.01, ***p < 0.001, ****p < 0.0001; ns, not significant. Original uncropped Western blot images are shown in File S1.

The role of FTO in lipid accumulation in THP1 macrophages was first determined. Ac‐LDL significantly increased LUR1 protein expression at a dose of 100 μg/mL, while FTO protein expression tended to decrease (Figure 1A–F). FTO was successfully overexpressed in THP‐1 macrophages by transfection with recombinant overexpression lentiviruses (OE‐FTO), and its effects on the accumulation of lipids in macrophages were investigated (Figure 1G–I). OE‐FTO obviously decreased the TC content in THP‐1 macrophages incubated with ac‐LDL (Figure 1L). ORO staining also revealed that FTO overexpression strongly decreased the number and volume of LDs in ac‐LDL‐treated THP‐1 macrophages (Figure 1J,K). On the other hand, the knockdown of FTO was induced in THP1 macrophages with small interfering RNA (siRNA), and siFTO‐1 was confirmed to achieve the best efficacy in FTO knockdown (Figure 1M–O). The knockdown of FTO dramatically increased the TC content in THP1 macrophages, which significantly increased the formation of intracellular LDs (Figure 1P–R). These data indicate that FTO plays a repressive role in the accumulation of lipids in macrophages.

### 3.2 FTO Inhibited Lipid Accumulation in Macrophages via LUR1

**FIGURE 2:**
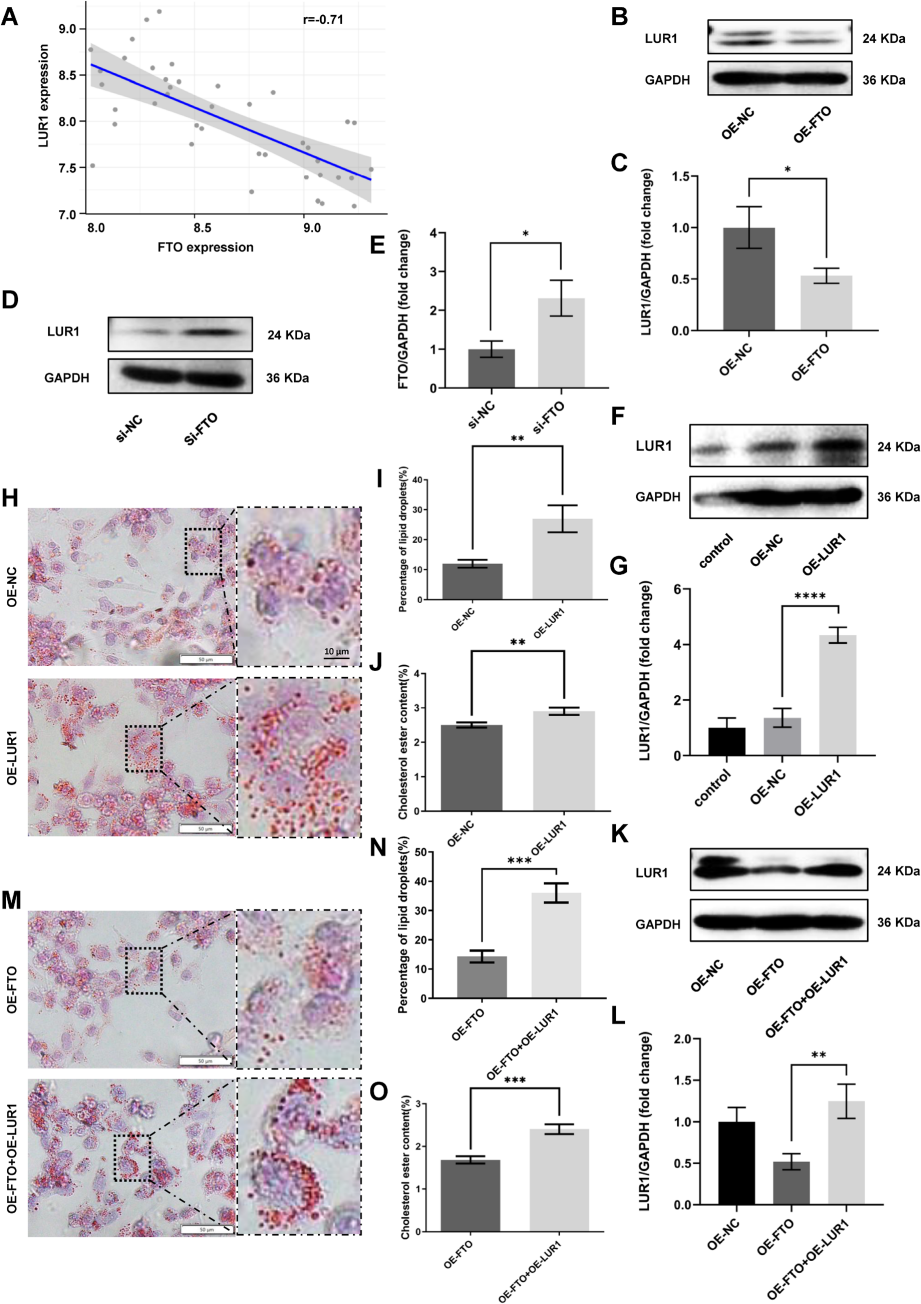
The crucial role of LUR1 in FTO‐inhibited lipid accumulation in macrophages. (A) The correlation between FTO and LUR1 was analysed in GEO datasets obtained from the Bioinfohome website. *R* = −0.71, *n* = 40 (GSE125771). (B, C) LUR1 protein expression in THP‐1 macrophages transfected with OE‐FTO for 48 h. (D, E) LUR1 protein expression in THP‐1 macrophages transfected with si‐FTO‐1 for 48 h. (F, G) LUR1 protein expression in THP‐1 macrophages transfected with OE‐LUR1 for 48 h. (H, I) Intracellular LDs stained with ORO in THP‐1 macrophages transfected with OE‐LUR1 for 48 h (40×; inset shows a magnified view of representative LDs; scale bars, 50 μm [original] and 10 μm [inset]). (J) Intracellular contents of TC measured by ELISA in THP‐1 macrophages transfected with OE‐LUR1 for 48 h. (K, L) LUR1 protein expression in THP‐1 macrophages transfected with OE‐FTO alone or combined with OE‐LUR1 for 48 h. (M, N) Intracellular LDs stained with ORO in THP‐1 macrophages transfected with OE‐FTO alone or combined with OE‐LUR1 for 48 h (40×; inset shows a magnified view of representative LDs; scale bars, 50 μm [original] and 10 μm [inset]). (O) Intracellular contents of TC measured by ELISA in THP‐1 macrophages transfected with OE‐FTO alone or combined with OE‐LUR1 for 48 h. All the results are expressed as the mean ± SD of three independent experiments in duplicate; *p < 0.05, **p < 0.01, ***p < 0.001, ****p < 0.0001; ns, nonsignificant. Original uncropped Western blot images are shown in File S1.

The relationship between FTO and LUR1 in the context of AS was bioinformatically analysed using the GEO database (https://www.ncbi.nlm.nih.gov/geo/#GSE125771), which revealed a strong negative correlation between FTO and LUR1 expression in human atherosclerotic plaques (*R* = −0.71) (Figure 2A). Further investigation was performed in THP‐1 macrophages, and Western blotting revealed a marked decrease in the expression of LUR1 protein in the OE‐FTO group compared with that in the OE‐NC group (Figure 2B,C), whereas LUR1 protein expression was upregulated in response to siFTO‐1 transfection (Figure 2D,E). These results demonstrated that FTO negatively regulates the expression of LUR1 in macrophages. LUR1 was overexpressed in THP‐1 macrophages through transfection with recombinant overexpression lentivirus (OE‐LUR1) to observe its role in lipid metabolism in THP‐1 macrophages, which was validated by Western blotting (Figure 2F,G). OE‐LUR1 markedly increased the TC content and promoted LD formation in THP‐1 macrophages (Figure 2H–J). Furthermore, when THP‐1 macrophages were cotransfected with OE‐FTO and OE‐LUR1, the LUR1‐suppressing and lipid‐lowering effects of OE‐FTO were reversed by the concomitant administration of OE‐LUR1, causing LUR1 upregulation accompanied by lipid accumulation in THP‐1 macrophages (Figure 2K–O). These findings suggest that FTO inhibits LUR1 expression to reduce lipid accumulation in macrophages.

### 3.3 FTO‐Catalysed m6A Demethylation Downregulated LUR1 Expression in a YTHDF1‐Dependent Manner

**FIGURE 3:**
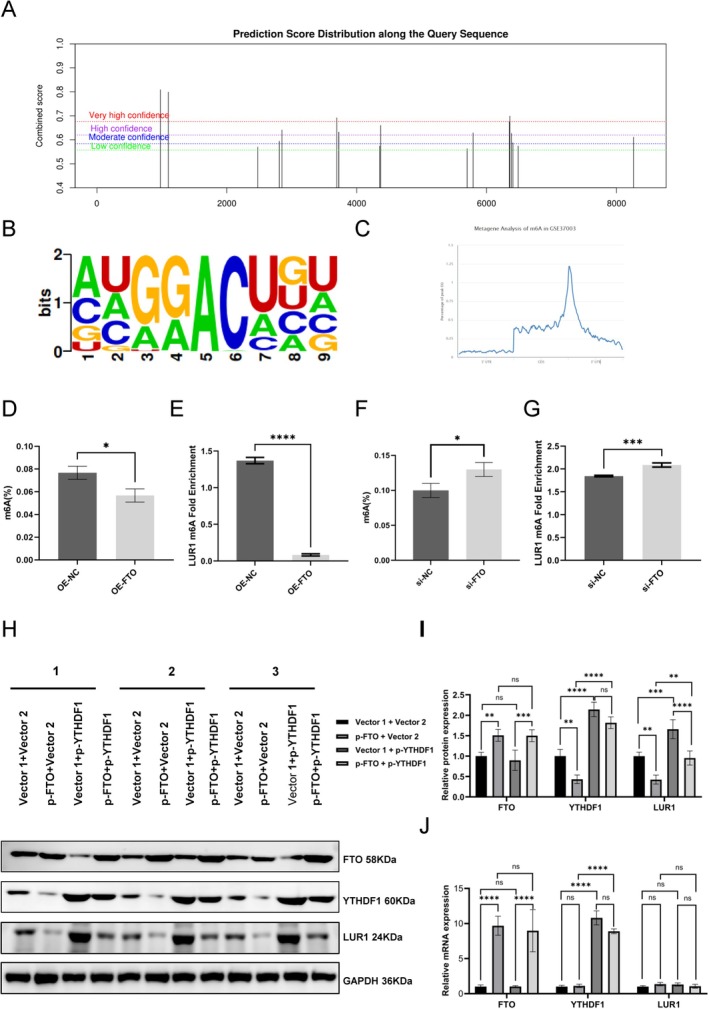
Effect of the demethylase FTO on m6A modification of the LUR1 mRNA strand and its downstream regulatory mechanism. (A) Potential m6A sites in LUR1 mRNA predicted by the SRAMP database. (B, C) Conserved m6A modification motifs analysed using the RMBase v2.0 database and metagene profiling. (D) Global m6A levels in total mRNA from THP‐1 macrophages transfected with OE‐FTO for 48 h. (E) m6A levels on LUR1 mRNA in THP‐1 macrophages transfected with OE‐FTO for 48 h. (F) Global m6A levels in total mRNA from THP‐1 macrophages transfected with si‐FTO for 48 h. (G) m6A levels on LUR1 mRNA in THP‐1 macrophages transfected with si‐FTO for 48 h. (H) Protein levels of FTO, YTHDF1, and LUR1 detected by Western blot analysis in THP‐1 macrophages transfected with p‐FTO, p‐YTHDF1, or their combination for 48 h. GAPDH served as the loading control. (I) Quantification of the relative protein expression corresponding to (H). (J) Relative mRNA levels of FTO, YTHDF1 and LUR1 determined by qRT–PCR in THP‐1 macrophages transfected as described in (H). All the results are expressed as the mean ± SD of three independent experiments in duplicate; *p < 0.05, **p < 0.01, ***p < 0.001, ****p < 0.0001; ns, not significant. Original uncropped Western blot images for Figure 3H are shown in File S3.

As a demethylase, FTO was speculated to change the m6A modification of LUR1 mRNA to modulate its expression. Given the conserved motif for m6A modification on the substrate mRNA strand, SRAMP (http://www.cuilab.cn/sramp) and RMBase v2.0 (https://rna.sysu.edu.cn/rmbase/index.php) were used to predict m6A sites on the LUR1 mRNA strand. We detected multiple modified m6A sites on LUR1 mRNA that were mostly close to the junction region between the exons and introns (Figure 3A–C). We quantified the global level of m6A in THP‐1 macrophages by using an m6A RNA methylation quantification kit. Quantitative detection revealed that the global level of m6A on total mRNA decreased significantly under OE‐FTO treatment (Figure 3D) but apparently increased in THP‐1 cells cultured with si‐FTO (Figure 3F). Moreover, independent MeRIP‐qPCR was conducted to verify the changes in m6A modification on the LUR1 mRNA strand. Consistent with the alteration of global m6A modification, FTO overexpression decreased m6A modification on LUR1 mRNA (Figure 3E), whereas FTO silencing promoted m6A modification (Figure 3G). These results demonstrate that FTO regulates LUR1 through m6A demethylation. Furthermore, we investigated whether FTO reduces LUR1 protein expression via the m6A reader YTHDF1 in THP‐1 macrophages. FTO overexpression decreased LUR1 protein levels, whereas YTHDF1 overexpression increased them (Figure 3H,I). Notably, compared with FTO overexpression alone, cotransfection of FTO and YTHDF1 (p‐FTO + *p*‐YTHDF1) significantly restored LUR1 protein expression, suggesting that FTO‐mediated demethylation attenuated the upregulation of LUR1 protein expression by YTHDF1. The levels of LUR1 mRNA remained unchanged across all the transfection groups (Figure 3J). Collectively, these findings indicate that FTO decreases LUR1 expression, primarily by attenuating YTHDF1‐dependent translational enhancement.

### 3.4 FTO‐Modulated LUR1 Participated in Aortic Lipid Deposition and AS

**FIGURE 4:**
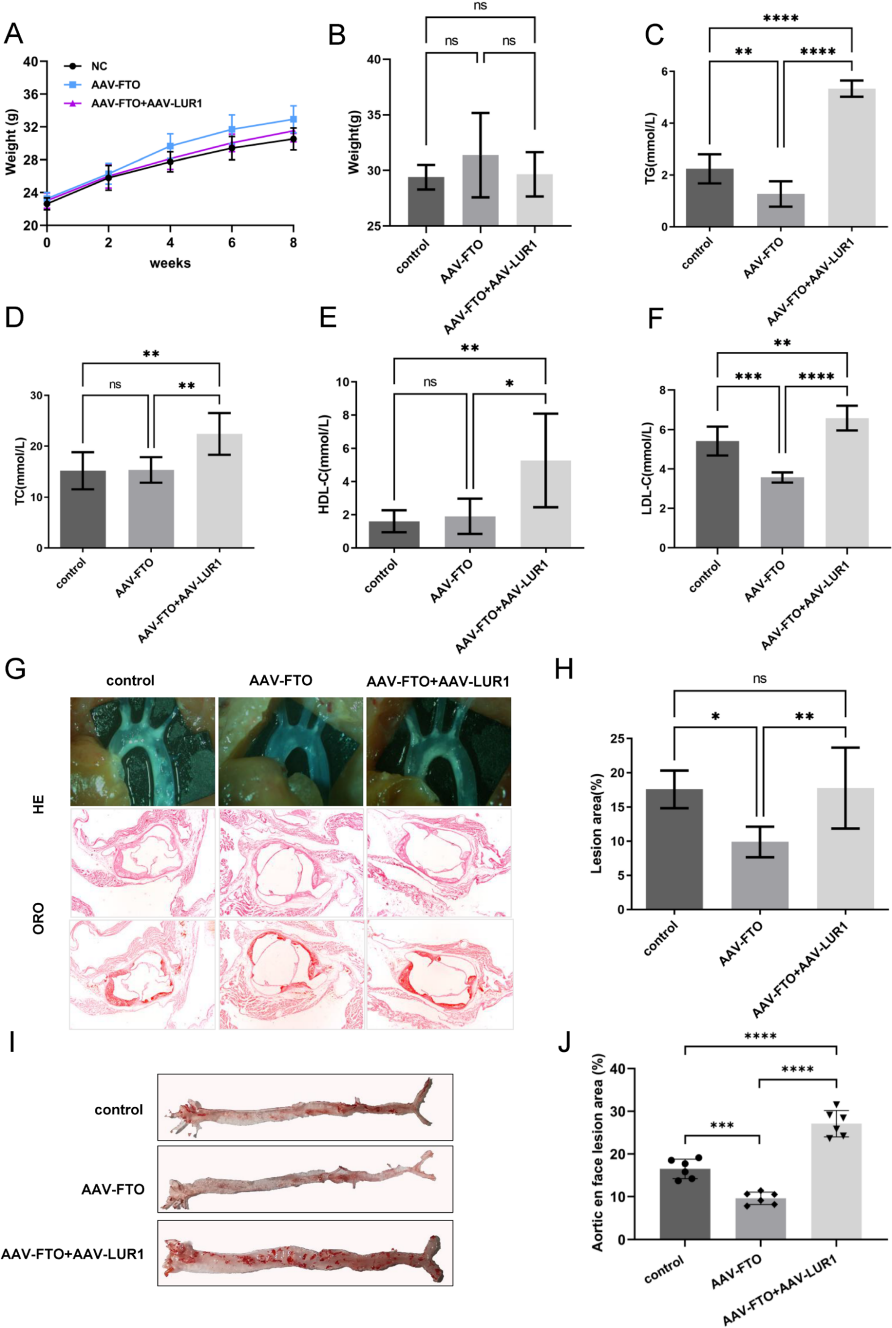
Plasma lipid profiles and aortic atherosclerotic lesions after treatment with FTO and LUR1 in vivo. (A) Body weights were measured every 2 weeks during animal treatment. (B) Body weights were measured at the end of the animal treatment. (C–F) Plasma levels of TG, TC, HDL‐C and LDL‐C in HFD‐fed apoE^−/−^ mice injected with AAV‐FTO alone or combined with AAV‐LUR1. (G) Stereomicroscopic observation (1.5×) and HE and ORO staining (4×) of the aortic walls of HFD‐fed apoE^−/−^ mice injected with AAV‐FTO alone or combined with AAV‐LUR1. (H) Quantification of atherosclerotic lesion areas in ORO‐stained aortic root cryosections. (I) Representative images of *en face* ORO staining of aortas from HFD‐fed apoE^−^/^−^ mice injected with AAV‐FTO alone or combined with AAV‐LUR1. (J) Quantification of the *en face* lesion area, expressed as a percentage of the total aortic surface area. Data are presented as the mean ± SD (*n* = 6 mice per group), *p < 0.05, **p < 0.01, ***p < 0.001, ****p < 0.0001; ns, not significant.

AAV‐FTO alone or combined with AAV‐LUR1 was injected into HFD‐fed apoE^−/−^ mice via the tail vein to regulate the expression of FTO and LUR1 in vivo. There were no alterations in body weight during the duration or at the end of the animal treatment, which excluded the effects of obesity and adeno‐associated viruses on atherogenesis (Figure 4A,B). Blood lipid tests revealed that compared with the control treatment, AAV‐FTO treatment mainly decreased the plasma levels of TG and LDL‐C. Simultaneous overexpression of FTO and LUR1 resulted in significant increases in the plasma levels of TG, TC, HDL‐C and LDL‐C (Figure 4C–F). Stereoscopic analysis revealed that the atherosclerotic plaques in the aortic arch and its three branches were distinctly alleviated by AAV‐FTO administration but exacerbated by AAV‐FTO + AAV‐LUR1 (Figure 4G). Naturally, the areas of atherosclerotic lesions and lipid deposition were reduced in the aortic sinuses of AAV‐FTO‐infected mice and markedly aggravated in mice injected with AAV‐FTO combined with AAV‐LUR1 (Figure 4G,H). Furthermore, the *en face* lesion area was significantly reduced in the AAV‐FTO group but notably increased in the AAV‐FTO + AAV‐LUR1 group, even exceeding the control level (Figure 4I,J). These observations revealed that FTO‐modulated LUR1 prevents lipid deposition and the development of AS in the aortic wall in vivo.

### 3.5 FTO Downregulated Aortic LUR1 Expression In Vivo

**FIGURE 5:**
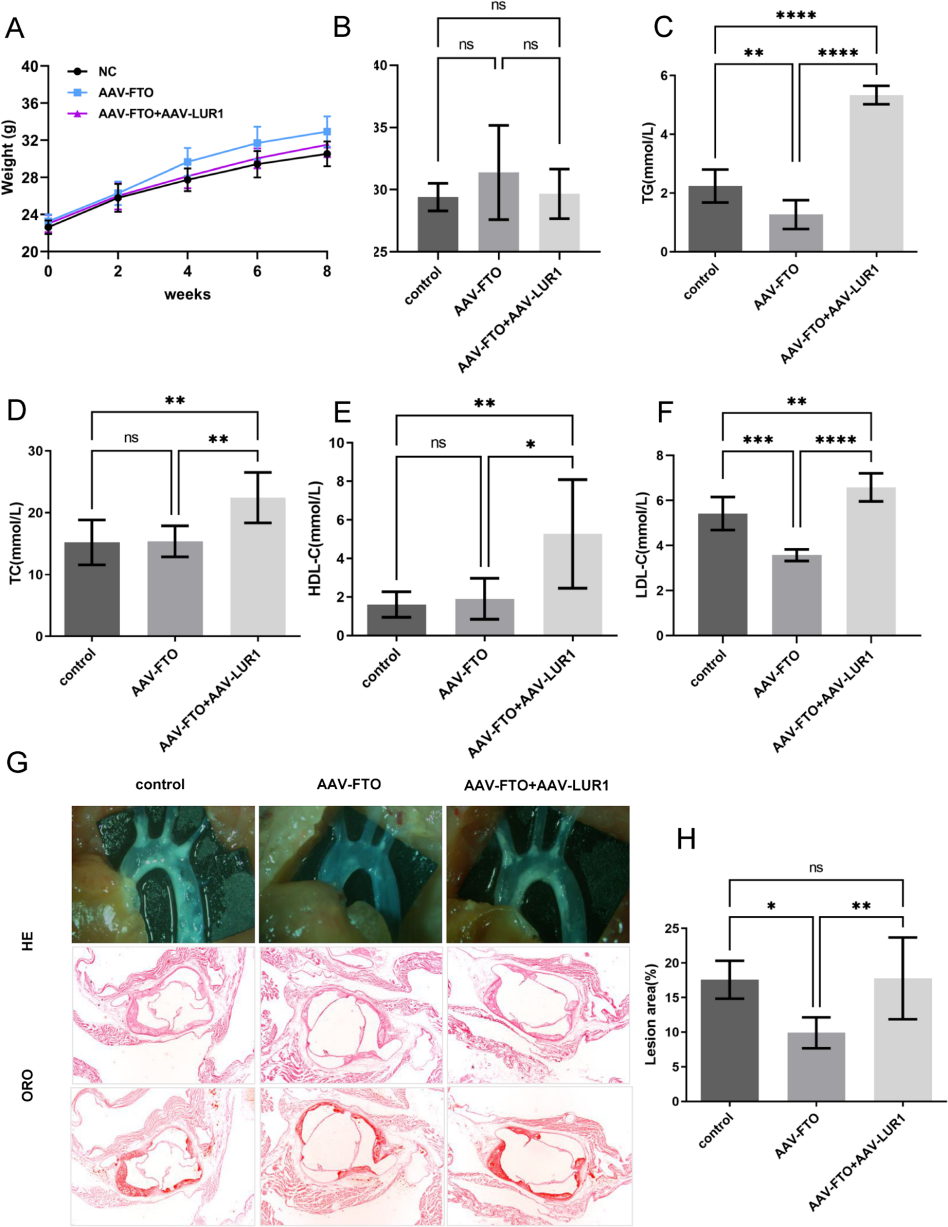
The expression of FTO and LUR1 in the aortic wall after treatment with FTO and LUR1 in vivo. (A, B) FTO protein expression in the aortic walls of HFD‐fed apoE^−/−^ mice injected with AAV‐FTO alone or combined with AAV‐LUR1. (C, D) The expression of LUR1 protein in the aortic walls of HFD‐fed apoE^−/−^ mice injected with AAV‐FTO alone or combined with AAV‐LUR1. The data are presented as the mean ± SD (*n* = 3 mice per group); *p < 0.05, **p < 0.01, ***p < 0.001, ****p < 0.0001; ns, not significant.

To further identify the inhibitory effect of FTO on aortic LUR1 expression in vivo, immunohistochemistry and immunofluorescence were used to evaluate changes in the expression of FTO and LUR1, respectively, in frozen aortic root sections. Immunohistochemical staining revealed successful changes in the expression of intracorporeal FTO in the model mice (Figure 5A,B). Immunofluorescence staining confirmed that the level of LUR1 protein in the aortic wall was markedly lower in the FTO‐overexpressing mice than in the control animals, but this effect was reversed by coinjection of AAV‐LUR1, which was in line with the results of the abovementioned cell experiments (Figure 5C,D). These findings suggest that intracorporeal FTO downregulates LUR1 expression in the aortic wall.

## 4 Discussion

**FIGURE 6:**
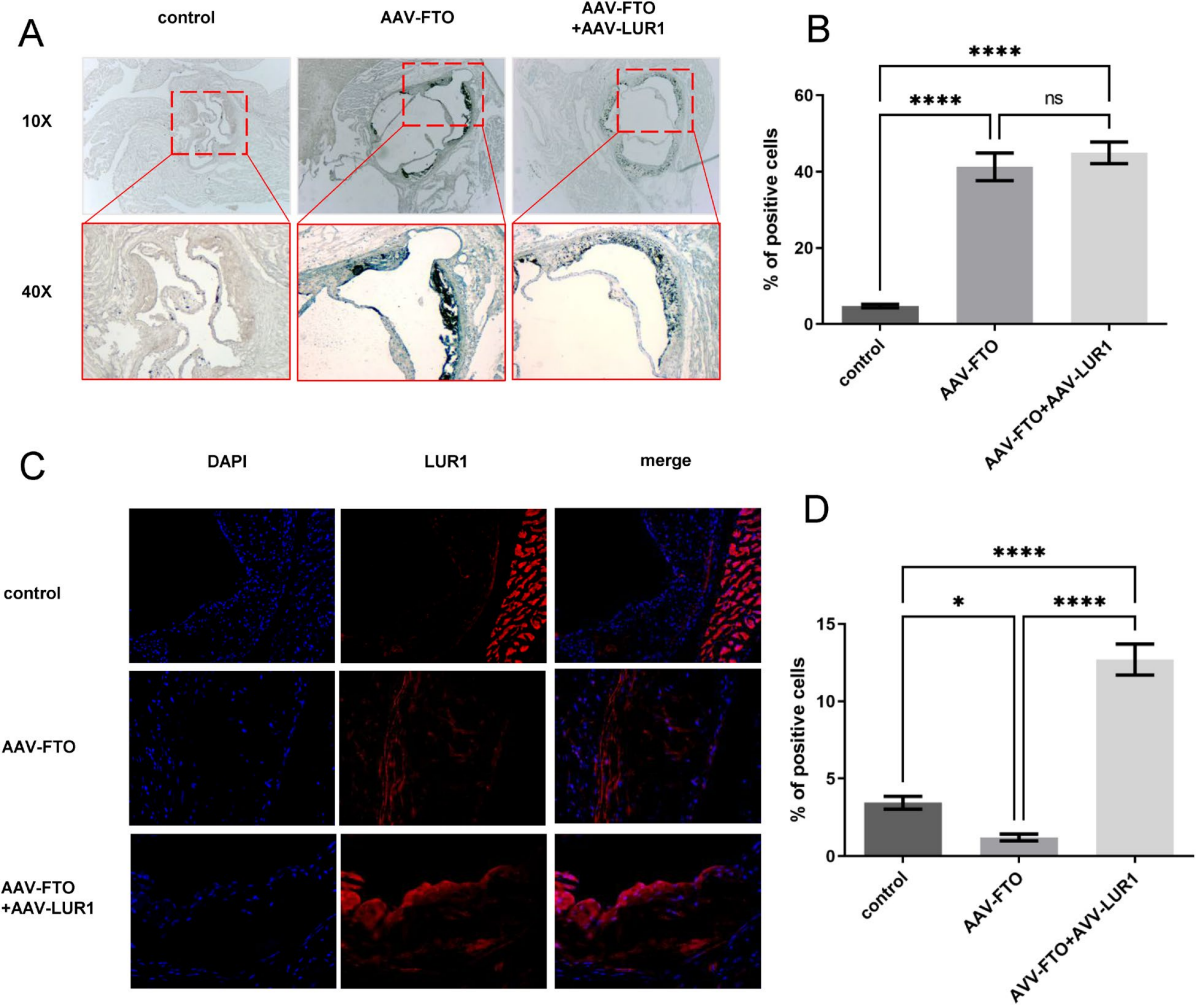
FTO‐erased m6A modification decreases macrophage LUR1 expression, thereby inhibiting lipid accumulation in macrophages and the occurrence of AS. As a demethylase, FTO‐catalysed demethylation of the LUR1 mRNA strand abates LUR1 expression and lipid accumulation in macrophages, suppressing foam cell formation and aortic lipid deposition and ultimately having a protective effect against AS in vivo.

The accumulation of lipids in macrophages and their subsequent transformation into foam cells represent crucial factors for the prognosis of AS [25]. Continuous ingestion of invading lipoproteins in the subendothelium accelerates the transformation of macrophages into foam cells and the development of AS [26]. The elimination of excessive intracellular lipids inhibits lipid accumulation in macrophages and the occurrence of AS in the aortic wall, and the inhibition of lipid accumulation has been a constant research hotspot in the prevention and treatment of AS [25, 26, 27]. Our study demonstrated that FTO obviously decreased the cellular contents of lipids in macrophages, which was abrogated by the simultaneous expression of LUR1. The expression of the new regulator of lipid metabolism LUR1 was prominently downregulated when FTO was overexpressed in macrophages. Mechanistic investigations revealed that FTO, a demethylase, apparently decreased the m6A modification of LUR1 mRNA, leading to a decrease in the protein level of LUR1 in macrophages. Plasma levels of TG and LDL‐C decreased in atherosclerotic model animals with FTO overexpression but were increased after the concurrent high expression of LUR1. FTO reduced lipid deposition and plaque area, which was also reversed by high LUR1 expression in the aortic wall. Aortic LUR1 levels decreased in response to FTO overexpression in vivo. This study preliminarily clarified that FTO‐erased m6A modification decreases LUR1 expression in macrophages, thereby inhibiting intracellular lipid accumulation and the occurrence of AS (Figure 6).

The new regulator LUR1 plays an important role in intracorporeal lipid metabolism, and determining its effect on proatherosclerotic lipid accumulation and the upstream mechanism controlling LUR1 expression and biological function in macrophages is important [6, 28]. LUR1 was shown to be related to hyperlipidemia in zebrafish and is speculated to be closely associated with lipid metabolism disorders in humans [7]. The deletion of LUR1 inhibited signal transduction of the SREBP pathway in Hap1 cells, Hepa1‐6 hepatoma cells, and rat primary hepatocytes [8]. The mRNA levels of the SREBP‐manipulated lipid‐related genes HMGCR, LDLR, SQLE and FASN were reduced, and the response to sterol deletion was largely decreased in Hap1‐*LUR1*‐KO cells [8]. Consistently, our research revealed that LUR1 strongly increased the lipid content in macrophages, facilitating intracellular lipid accumulation and foam cell formation. LUR1 overexpression increased plasma levels of proatherosclerotic lipids in vivo, resulting in aortic lipid deposition and the development of AS. Importantly, our in vivo co‐overexpression data revealed the dominant pro‐atherogenic potency of LUR1. Its overexpression not only abrogated FTO‐mediated atheroprotective effects but also drove disease severity beyond baseline, underscoring the therapeutic necessity of concurrently targeting both FTO and its downstream node, LUR1. The downstream pathway of LUR1‐regulated lipid metabolism in macrophages was not investigated in this study. LUR1 has been reported to catalyse the cleavage of the prodomain from S1P precursors to accelerate the maturation and Golgi localization of S1P and to activate the SREBP pathway, which probably also affects lipid metabolism in macrophages [9]. Instead of the downstream pathway targeted by LUR1, our group innovatively explored the upstream mechanism that modulates LUR1 expression and biological function in macrophages. Many m6A modification sites were found on the LUR1 mRNA strand, indicating the regulatory effect of m6A modification on the expression and activity of LUR1. Our experimental evidence further confirmed that FTO‐erased m6A modification downregulated LUR1 expression and lipid content and alleviated lipid accumulation and aortic lipid deposition in macrophages, providing a prospective strategy for AS treatment.

FTO functions as a generalist and is involved in various aspects of lipid metabolism [29, 30, 31]. The overexpression of FTO reduced the cellular content of oxidized LDL and the scavenger receptor CD36 in RAW264.7 macrophages, which inhibited the formation of foam cells in the early stage of AS and the progression of more complex vulnerable plaques [32]. FTO attenuated lipid uptake partly by decreasing PPARγ activity and increasing cholesterol efflux through AMPK activation in RAW264.7 macrophages, thereby inhibiting aortic lipid deposition and decreasing the size of atherosclerotic plaques in apoE^−/−^ mice [32]. These previous investigations treated FTO as only a transcription factor or a phosphatase or did not explore its regulatory mechanism in terms of lipid metabolism [33, 34]. In our study, FTO downregulated LUR1 expression and inhibited lipid metabolism in THP‐1 macrophages, substantially reduced the presence of intracellular LDL and suppressed the formation of foam cells. In vivo experiments validated the protective role of FTO in the plasma lipid profile, aortic lipid deposition and atherosclerotic lesions. In contrast to the existing studies, we further investigated novel mechanisms through which FTO manipulates LUR1 expression in macrophages. FTO was confirmed to function as an m6A demethylase, catalysing the demethylation of the LUR1 mRNA strand and substantially reducing the expression of LUR1 in macrophages, revealing new downstream targets and mechanistic insights into the role of FTO in lipid metabolism regulation and AS therapy.

However, several limitations in this study need to be noted and further discussed. First, the differential expression patterns of FTO and LUR1 under 50 versus 100 μg/mL ac‐LDL treatments remain mechanistically unexplained. However, this discrepancy does not compromise our overall conclusions, as all functional validation and rescue experiments were performed under the 100 μg/mL condition, a well‐established and commonly used concentration for inducing foam cell formation. Second, the exploration of the role of FTO‐controlled lipid metabolism in macrophages was weak and superficial in this study, especially because FTO functions as a demethylase of m6A modification [17, 35]. FTO may potentially erase m6A modification on the mRNA strands of other lipid‐related genes in addition to LUR1. The comprehensive efficacy and detailed mechanisms of FTO warrant further exploration in terms of macrophage lipid metabolism and atherogenesis. Third, although the present study confirmed successful FTO overexpression in the aortic wall, future studies are warranted to systematically evaluate effective FTO overexpression in other metabolic tissues, including the liver, adipose tissue, heart and lungs, to fully elucidate its tissue‐specific distribution and potential systemic metabolic effects. Fourth, FTO is known to increase body weight and accelerate the development of obesity [32, 36]. Unexpectedly, the body weights of HFD‐fed apoE^−/−^ mice overexpressing FTO did not significantly increase compared with those of apoE^−/−^ mice fed only an HFD, possibly because the strong preference for a HFD masked the impact of FTO on body weight. Finally, FTO has been demonstrated to aggravate lipid deposition in the liver, the adipogenesis of preadipocytes and the formation of white adipose tissue but also, paradoxically, to decrease macrophage lipid accumulation and aortic plaque formation, leading to a therapeutic dilemma of intracorporeal FTO intervention [37]. It is necessary to develop a reliable strategy to achieve sufficient positive efficacy but to avoid side effects associated with internal FTO modulation in the future.

## 5 Conclusions

In summary, our study preliminarily revealed that FTO‐catalysed m6A demethylation decreased LUR1 expression, lipid accumulation in macrophages, and foam cell formation. In vivo research confirmed that FTO‐erased m6A modification reduced proatherosclerotic plasma lipid levels, lipid deposition, and plaque area in the aortic wall. Importantly, these protective effects were abolished by concurrent LUR1 overexpression, corroborating LUR1 as the pivotal downstream mediator of the FTO–m^6^A axis. The epigenetic modulatory effect of FTO on LUR1 was confirmed to have therapeutic value in atherosclerotic cardiovascular diseases. The overall influences and detailed mechanisms of FTO and LUR1 on lipid metabolism and AS occurrence require further investigation and evaluation. Intervention strategies involving intracorporeal FTO and LUR1 need to be developed urgently, and their subtle side effects need to be bypassed by the academic community. A comprehensive and in‐depth understanding of the role of FTO and LUR1 in lipid metabolism regulation will hopefully provide a new avenue for the clinical treatment of atherosclerotic cardiovascular diseases.

## Author Contributions

Yun‐Cheng L.V.: conceptualization, methodology, formal analysis, funding acquisition, writing – review and editing. Xiang‐Yang Tang: conceptualization, investigation, formal analysis, writing – original draft, writing – review and editing. Le Zhou: investigation, formal analysis. Shu‐Jun Li: investigation. Shao‐Xiang Zhang: investigation. Yue‐Ying Yuan: investigation, formal analysis. All the authors reviewed the manuscript.

## Funding

This study was supported by the National Natural Science Foundation of China (82160098), the Natural Science Foundation of Guangxi Zhuang Autonomous Region (2024JJA141030) and the Guangxi Key Clinical Specialty (GuiWeiYiFa [2023] No. 26).

## Ethics Statement

All animal experiments were carried out in strict accordance with the procedures reviewed and approved by the Laboratory Animal Ethics Committee of Guilin Medical University (Approval No. GLMC‐IACUC‐20251093).

## Consent

The authors have nothing to report.

## Conflicts of Interest

The authors declare no conflicts of interest.

## Supporting information


**File S1:** Original uncropped Western blot images for Figures 1 and 2.

**File S3:** Original uncropped Western blot image for Figure 3H.

## Data Availability

All relevant data are available within the manuscript. Original uncropped Western blot images are provided in Files S1 and S3. Additional raw data are available from the corresponding author upon reasonable request.
