# Supplementary material for: FTO‐Catalysed Demethylation of LUR1 mRNA Suppresses Macrophage Lipid Accumulation and Aortic Atherosclerosis
Source: J Cell Mol Med. 2026 Jun 11;30(11):e71234. doi: 10.1111/jcmm.71234 (PMC13259957; doi:10.1111/jcmm.71234)

Supplementary Material (1)

Fig.1A

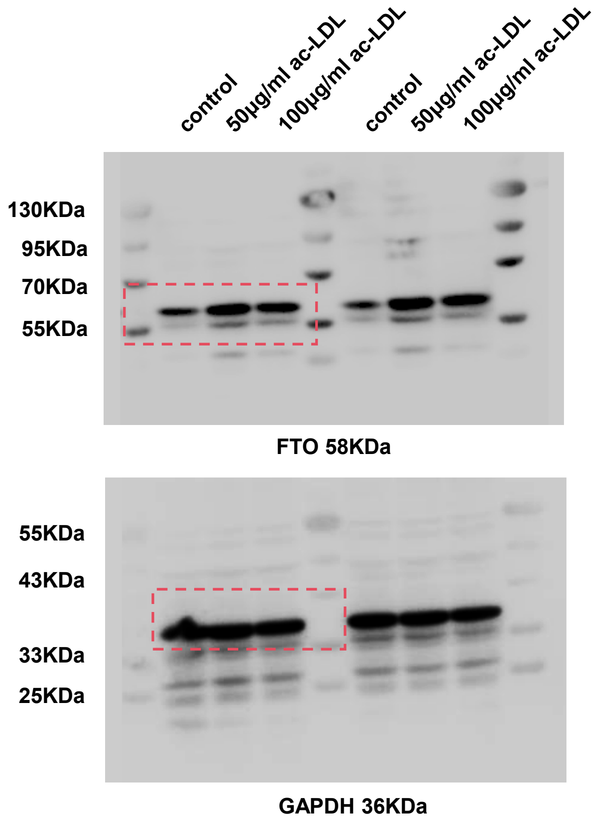

Fig.1B

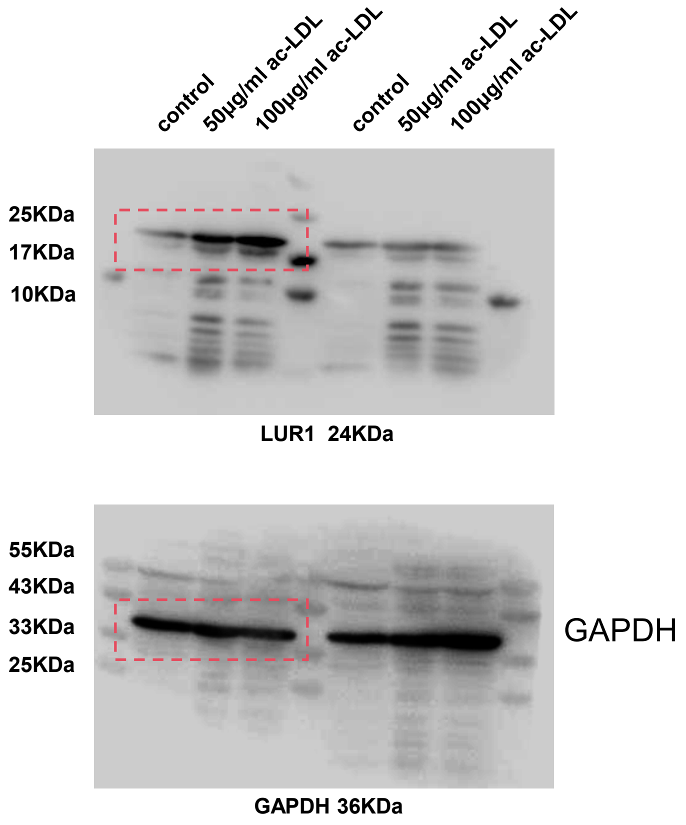

Supplementary Material (2)

Fig.1G

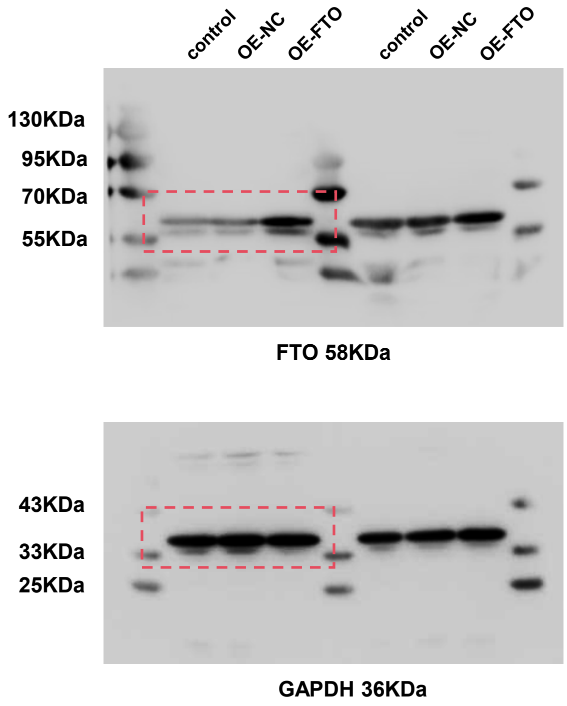

Fig.1M

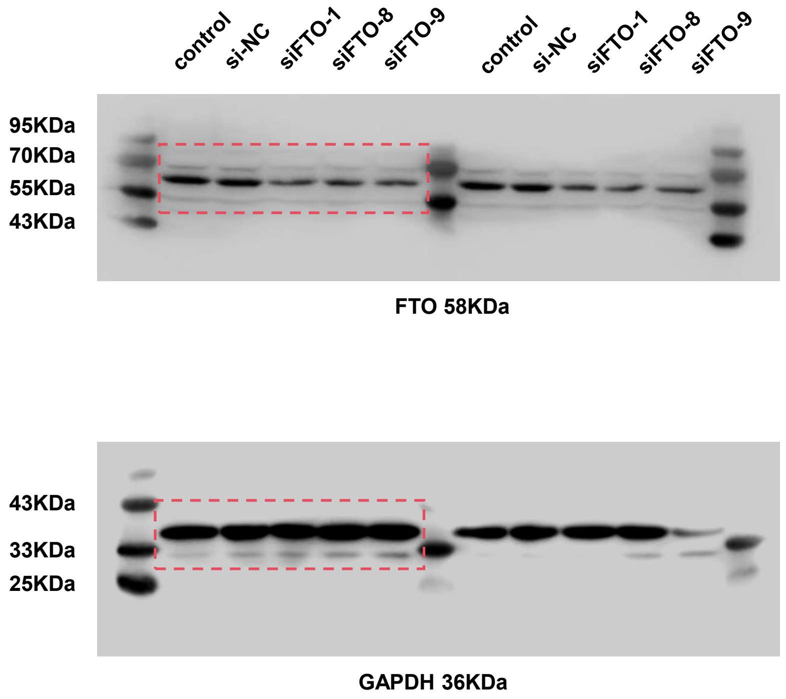

Supplementary Material (3)

Fig.2B

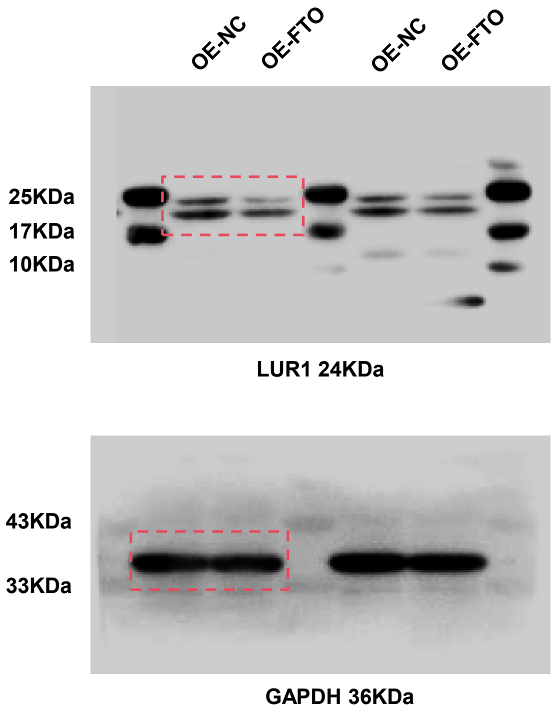

Fig.2D

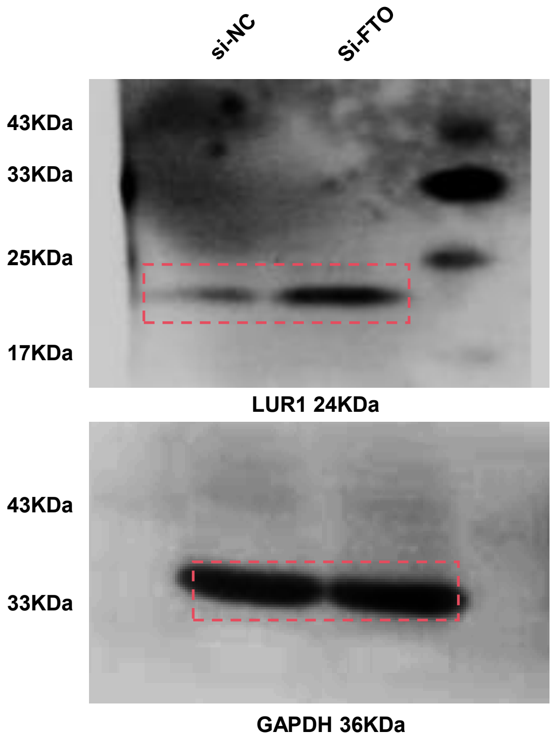

Supplementary Material (4)

Fig.2F

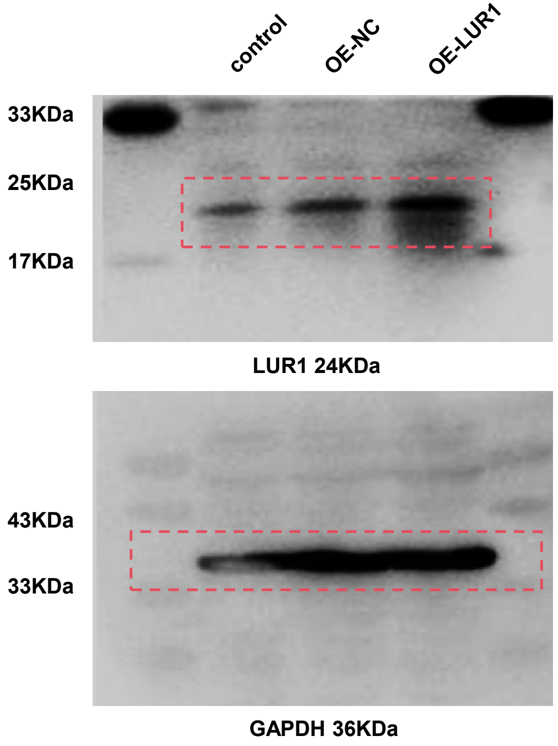

Fig.2K

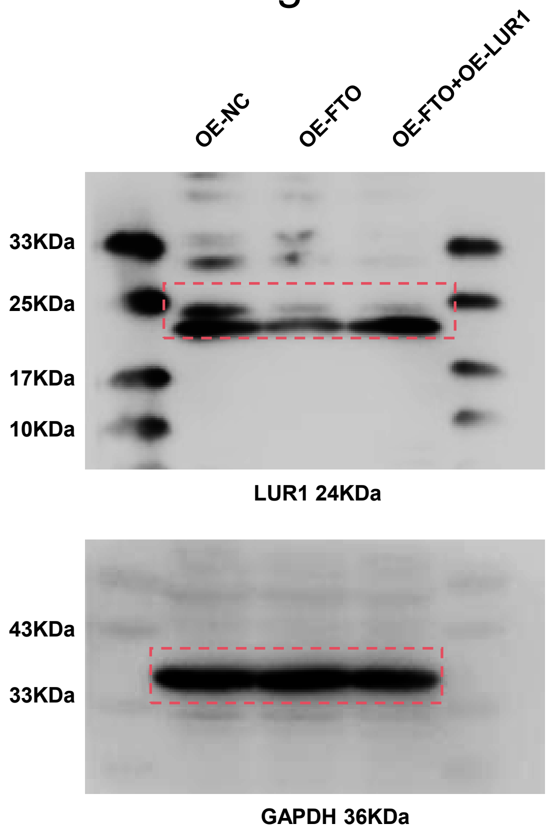

Supplement: Supplementary file 1 — File S1: Original uncropped Western blot images for Figures 1 and 2. [file JCMM-30-e71234-s001.pdf]
