# Supplementary figures and images for: FTO‐Catalysed Demethylation of LUR1 mRNA Suppresses Macrophage Lipid Accumulation and Aortic Atherosclerosis
Source: J Cell Mol Med. 2026 Jun 11;30(11):e71234. doi: 10.1111/jcmm.71234 (PMC13259957; doi:10.1111/jcmm.71234)

Original full-length blots for Figure 3H

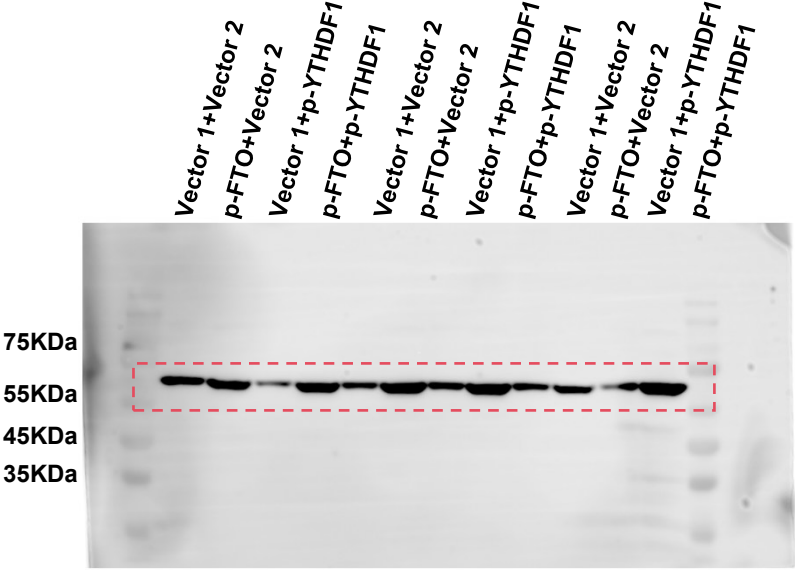

FTO 58KDa

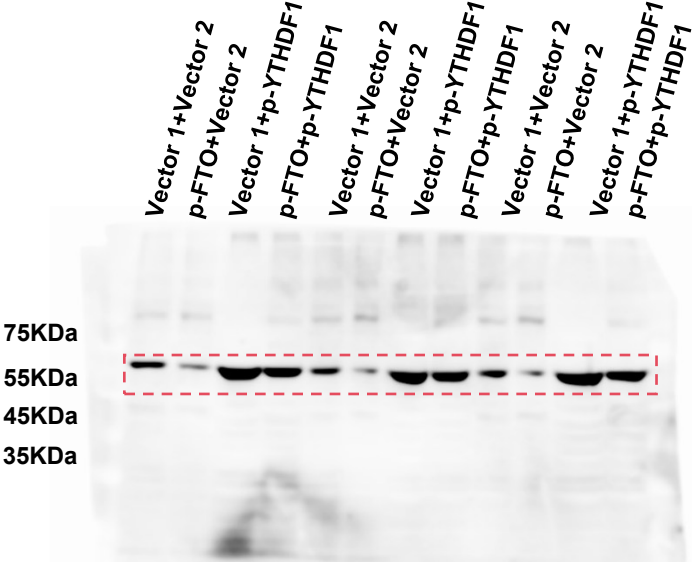

YTHDF1 60KDa

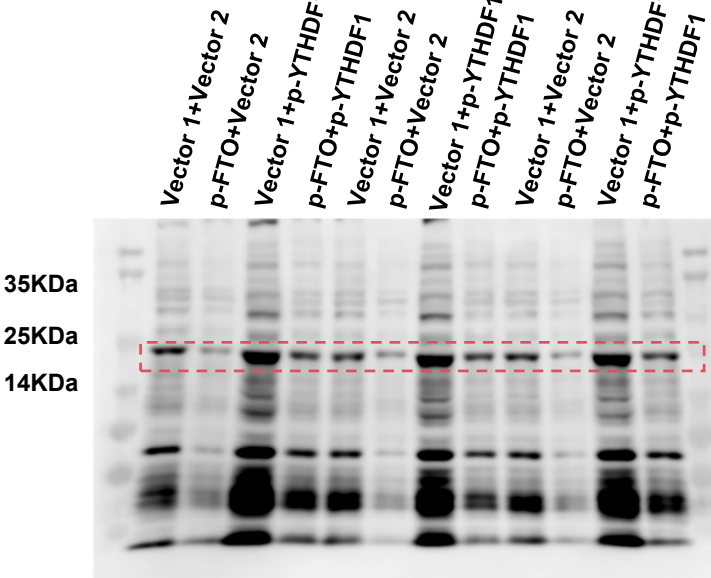

LUR1 24KDa

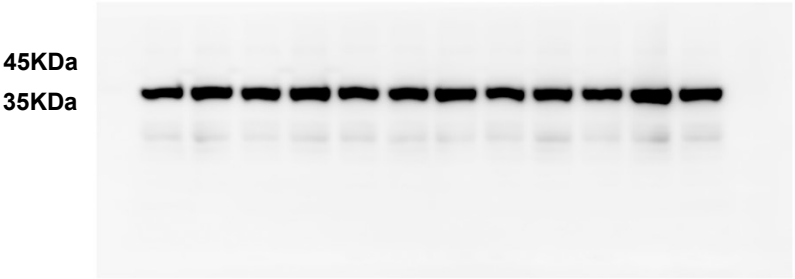

GAPDH 36KDa

Supplement: Supplementary file 2 — File S3: Original uncropped Western blot image for Figure 3H. [file JCMM-30-e71234-s002.pdf]
